# On the genetic basis of the effect of *Spiroplasma* on the male reproductive fitness of *Glossina fuscipes fuscipes*

**DOI:** 10.1371/journal.ppat.1010442

**Published:** 2022-04-04

**Authors:** Edward Oropeza-Rodriguez, Bryan D. Clifton, José M. Ranz

**Affiliations:** Department of Ecology and Evolutionary Biology, University of California Irvine, Irvine, California, United States of America; Ecole Polytechnique Federale de Lausanne, SWITZERLAND

Son and colleagues recently reported [1] the phenotypic effects of the infection of the endosymbiont *Spiroplasma* in the tsetse fly *Glossina fuscipes fuscipes*, a major disease vector in humans. The authors examined the impact of *Spiroplasma* on the reproductive biology of *G*. *f*. *fuscipes*, showing that infection by the pathogen modifies sex-biased expression in reproductive tissues, ultimately affecting female fecundity as well as sperm motility and competitiveness among other phenotypes. The effect on male reproductive biology is presented in connection to the Vector Base gene ID GFUI025244, *i*.*e*., a presumed ortholog to the *Drosophila melanogaster* coding gene *Sperm-specific dynein intermediate chain* (*Sdic*). In fact, the interpretation of the results is heavily reliant on the functional and phenotypic attributes of *Sdic* in *D*. *melanogaster*.

**Fig 1:**
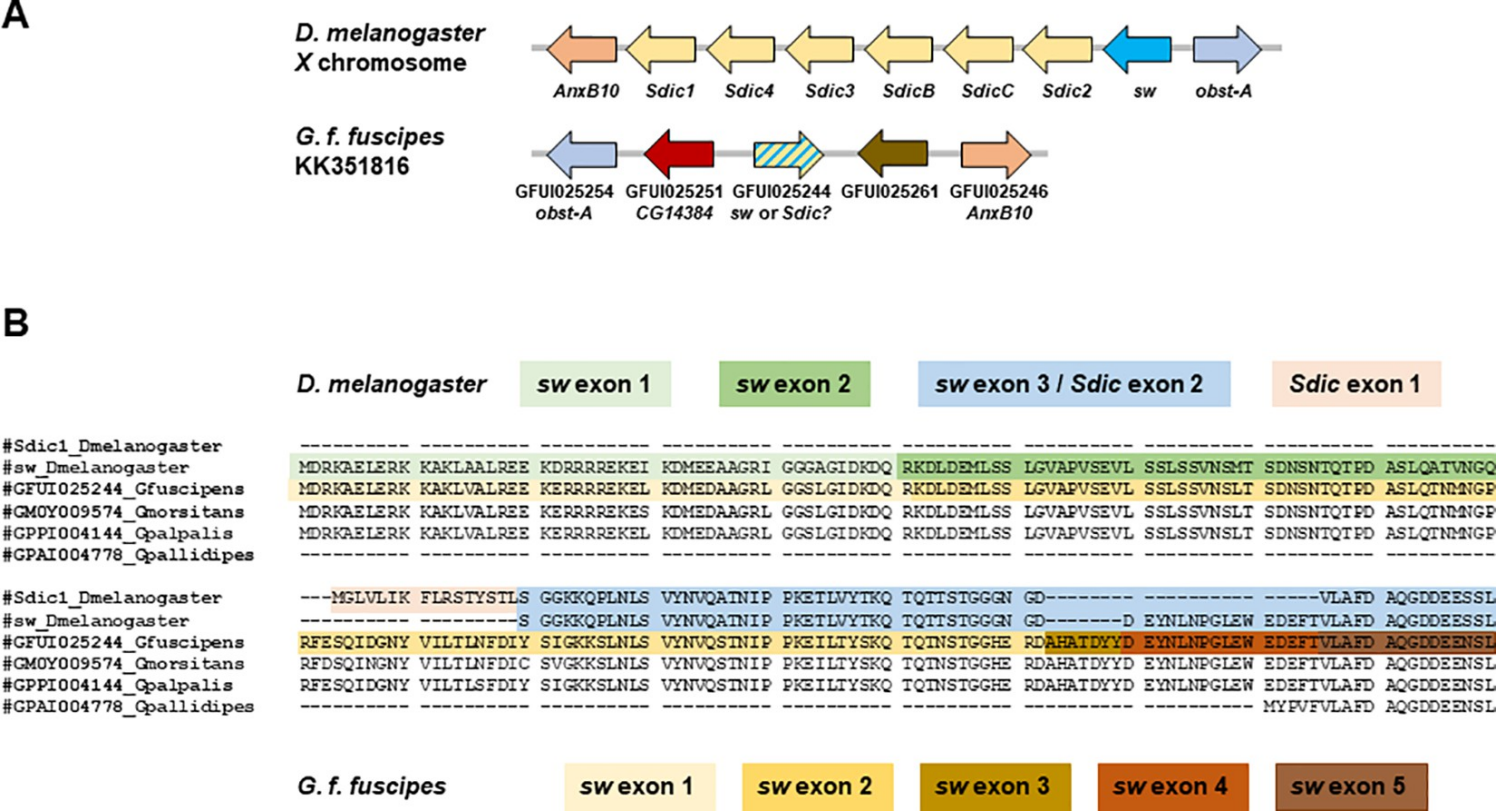
The ortholog to the *G*. *f*. *fuscipes* gene GFUI025244 in *D*. *melanogaster* is *sw* and not *Sdic*. (A) Gene organization surrounding *Sdic* and *sw* in *D. melanogaster* according to the annotation of the Berlin genome assembly for the reference strain ISO-1 [5] compared to that around the gene GFUI025244 in the supercontig KK351816 of the *G*. *f*. *fuscipes* genome assembly 3.0.2 (GenBank accession: GCA_000671735.1). For *G*. *f*. *fuscipes*, the name of the presumed ortholog in *D*. *melanogaster* is also provided. Gene size and spacing are not to scale. Based on the current assembly of the region, only one of the two genes, *Sdic* or *sw*, is present in *G*. *f*. *fuscipes*. (B) Sequence alignment of the *D*. *melanogaster* proteins Sdic and sw, and the protein predicted by VectorBase to correspond to Sdic in *G*. *f*. *fuscipes* and three close relatives. Only the first 200 amino acids from the N-terminus are shown. As a Sdic protein of *D*. *melanogaster*, only the product encoded by the paralog *Sdic1* in the reference strain ISO-1 is included in the alignment; all encoded proteins by *D*. *melanogaster Sdic* paralogs have an identical 5’ termini [5]. The amino acid sequence encoded by different exons of the genes *sw* and *Sdic* in *D*. *melanogaster* and *G*. *f*. *fuscipes* are color coded. The gene ID in each *Glossina* species is indicated before the species name. The presumed Sdic protein in a fourth species, *G*. *austeni*, was omitted as it is fragmented into three partial ORFs. As shown in the alignment, the encoded protein by GFUI025244 exhibits high sequence identity with the entire sw protein of *D*. *melanogaster*, which is not the case in relation to the Sdic1 protein as it is defective for the residues coded by the first two exons of *sw*.

In *D*. *melanogaster*, *Sdic* is a defective duplicate of the parental gene *short wing* (*sw*) [2], which encodes a cytoplasmic dynein intermediate chain [3] that acts as a subunit in protein complexes called dyneins–a class of molecular motors responsible for microtubule-based motility in the cell [4]. Expression profiling of *Sdic* showed that it is primarily expressed in male testes [2] but also in ovaries and other somatic tissues of both sexes [5]. Knockout experiments revealed that *Sdic* impacts sperm performance in competitive settings [6], likely through enhancing sperm displacement or retention ability in the sperm storage organs of the female [7]. *Sdic* has undergone multiple tandem duplications [8], forming a cluster on the *X* chromosome, which is flanked by the genes *sw* and *obst-A* upstream, and by *AnxB10* and *CG9581* downstream (Fig 1A). The Sdic proteins encoded by different paralogs are highly conserved except for a stretch at their carboxyl termini [5,8]. Crucially, *Sdic* has been found in all strains of *D*. *melanogaster* examined so far, but not in any other *Drosophila* or insect species [2,5,9]. Therefore, *Sdic* is a *D*. *melanogaster*-specific gene, conflicting with its assumed presence in *G*. *f*. *fuscipes* [1].

To clarify this disparity, we retrieved the ~16kb of genomic sequence centered around the presumed ortholog of *Sdic* in *G*. *f*. *fuscipes* (GFUI025244), which resides in the supercontig KK351816 (Fig 1A). As confirmed by BLASTP homology searches against *D*. *melanogaster*, this genomic fragment includes the orthologs to the coding genes *obst-A* (GFUI025254; E-value = 10^−106^) and *AnxB10* (GFUIO25246; E-value = 10^−143^) as outermost genes. According to the annotation in Vector Base, there is one additional coding gene immediately upstream (GFUI025251) and another one downstream (GFUIO25261) of GFUI025244. Reciprocal best match searches using BLASTP revealed that GFUI25251 is a likely ortholog of *CG14384* (E-value = 0), a gene located on the *3*L of *D*. *melanogaster*, while GFUI025261 does not have any significant match, an observation that holds when other Diptera are considered. Surprisingly, there is no annotation in Vector Base that corresponds to the ortholog of the gene *sw*, an essential gene in *D*. *melanogaster* [3]. In order to discard that the *sw* ortholog could be located elsewhere in the genome of *G*. *f*. *fuscipes*, we performed BLASTP searches against *G*. *f*. *fuscipes*. We found one main significant blast hit involving GFUI025244 (E-value = 10^−143^), *i*.*e*., the gene model annotated as the ortholog to *Sdic*, and two secondary hits associated with additional dynein intermediate chain encoding genes in *D*. *melanogaster* (GFUI019652, E-value = 10^−18^, the putative ortholog to *CG1571*; GFUI019660, E-value = 10^−10^, the putative ortholog to *Dnai2*). To discard that this region of the *G*. *f*. *fuscipes* genome was misassembled, thus providing a distorted reconstruction of the region where the putative *Sdic* ortholog could reside, we replicated the same analyses in the closely related species *G*. *austeni*, *G*. *morsitans*, *G*. *palpalis*, and *G*. *pallidipes*, finding identical results. This outcome directly challenges that the ortholog to GFUI025244 in *D*. *melanogaster* is *Sdic* (Fig 1A).

We then aligned the proteins encoded by the presumed *Sdic* in *G*. *f*. *fuscipes* and closely related species, as well as *Sdic1* and *sw* of *D*. *melanogaster* (Fig 1B). Except for *G*. *pallidipes*, species that exhibits a 180 amino acid residue deletion at the 5’ terminus, the protein sequence encoded by the presumed ortholog to *Sdic* is virtually identical across *Glossina* species. For alignable intervals, the encoded proteins in the *Glossina* species exhibit sequence identity values of 86.3–86.7% along their entire length with the protein sw of *D*. *melanogaster*. In contrast, the sequence identity of these proteins with that of Sdic of *D*. *melanogaster*, although also high (79.3–83.4%), is essentially absent across the 123 amino acid residues at the 5’ terminus. In *D*. *melanogaster*, the Sdic protein also lacks the 100 residues encoded by the first two exons of the sw protein. Notably, the amino acid residues encoded by the first exon of *Sdic*, which is conserved across all *Sdic* paralogs and unique to *Sdic*–therefore absent from *sw*–[5], are also lacking in the presumed Sdic proteins from the *Glossina* species. Collectively, these results strongly suggest that Son and colleagues [1] misidentified the ortholog to *sw* as the ortholog to *Sdic*, following the existing annotation in Vector Base.

*Spiroplasma* infection preferentially affects the expression of sex-biased genes in reproductive tissues of both males and females of *G*. *f*. *fuscipes* [1]. In relation to male reproductive fitness, the transcript of the putative ortholog to *Sdic*, as assumed by the authors [1]–here shown to be actually *sw*–, is particularly abundant in the spermathecae of females that mated with males free of *Spiroplasma* compared to equivalent matings involving infected males. In stark contrast, no differences in expression levels for the putative ortholog to *Sdic* were found in the male reproductive tract of infected versus noninfected males. Subsequent phenotypic tests determined that although the number of stored sperm in females did not differ when mated with infected versus noninfected males, the fill with sperm of the spermatheca decreased in infected females compared to noninfected females, and the beating frequency of the sperm from infected males was significantly lower compared to that of the sperm from noninfected males. Although conspicuous, these observations are merely correlative. In *D*. *melanogaster*, neither *sw* nor *Sdic* are exclusively expressed in sperm cells as expression has been documented in female and somatic tissues [5,10]. Additionally, it is not clear from the data presented whether the other genes expressed in the sperm are also downregulated in the spermatheca of females that mated with infected males. Therefore, any impaired sperm motility could be the result of other affected biological processes and not of one necessarily dependent on the *sw* ortholog.

As part of its general role in cytoplasmic functions in *D*. *melanogaster*, the protein sw interacts with the p150 subunit of the dynactin protein complex through the 5’ terminus and with the dynein light and heavy chains of the cytoplasmic dynein complex through the 3’ terminus [3]. The role of this protein in the sperm remains uncharacterized [11]. Therefore, even if the *sw* ortholog is the most impacted gene by *Spiroplasma* infection in *G*. *f*. *fuscipes*, it is unclear how this could affect sperm motility. Further, and in relation to the impact of *Spiroplasma* on reducing male fertility in *G*. *f*. *fuscipes*, which could have valuable consequences for vector control, several experiments should be performed: (i) to characterize the precise role of the ortholog to *sw* compared to other dynein subunit-encoding genes; (ii) to quantify any male fertility reduction in competition settings [12]; and (iii) to determine whether the ortholog to *sw* is essential, as it is in *D*. *melanogaster*, or at least relevant for overall organismal fitness. Particularly this latter point is crucial as it is unclear what is the true probability of success of a potential intervention that relies on the use of *Spiroplasma* infected males as this type of male might have a substantially lower fitness compared to uninfected males due to an impaired functionality of the *sw* ortholog in *G*. *f*. *fuscipes*.
